# Exploring the Effectiveness of Acyclovir against Ranaviral Thymidine Kinases: Molecular Docking and Experimental Validation in a Fish Cell Line

**DOI:** 10.3390/life14091050

**Published:** 2024-08-23

**Authors:** Richárd Rácz, Ákos Gellért, Tibor Papp, Andor Doszpoly

**Affiliations:** HUN-REN Veterinary Medical Research Institute, H-1143 Budapest, Hungary; racz.richard@semmelweis.hu (R.R.); gellert.akos@vmri.hun-ren.hu (Á.G.); papp.tibor@vmri.hun-ren.hu (T.P.)

**Keywords:** acyclovir, molecular docking, antiviral effect, European catfish virus, Frog virus 3, Ictalurid herpesvirus 2

## Abstract

The effectiveness of acyclovir, a selective anti-herpesvirus agent, was tested both in silico and in vitro against two ranaviruses, namely the European catfish virus (ECV) and Frog virus 3 (FV3). ECV can cause significant losses in catfish aquaculture, while FV3 poses a risk to vulnerable amphibian populations. The genome of ranaviruses encodes thymidine kinases (TKs) similar to those of herpesviruses. Molecular docking simulations demonstrated that the acyclovir molecule can bind to the active sites of both investigated viral TKs in an orientation conducive to phosphorylation. Subsequently, the antiviral effect of acyclovir was tested in vitro in Epithelioma Papulosum Cyprini (EPC) cells with endpoint titration and qPCR. Acyclovir was used at a concentration of 800 µM, which significantly reduced the viral loads and titers of the ranaviruses. A similar reduction rate was observed with Ictalurid herpesvirus 2, which was used as a positive control virus. These promising results indicate that acyclovir might have a wider range of uses; besides its effectiveness against herpesviruses, it could also be used against ranavirus infections.

## 1. Introduction

Acyclovir is an anti-herpesvirus agent [1,2] that is an acyclic analog of guanosine [3]. Acyclovir is generally used to treat infections caused by herpes simplex viruses (HSV-1 and HSV-2) and varicella-zoster virus (VZV). Acyclovir has been widely used in medication for the past four decades; however, it has also been shown to have a cytotoxic effect [4,5,6].

Its selective action against virus-infected cells is mediated through a series of well-defined steps that limit its impact on healthy cellular functions. Upon its administration, acyclovir is absorbed and preferentially taken up by cells infected with herpesvirus. Once inside the host cells, acyclovir is selectively phosphorylated into its monophosphate metabolite (ACV-MP) by viral kinases and its antiviral activity employed in this form [7,8,9]. Intracellular guanylate kinase enzymes transform the monophosphate into the diphosphate form (ACV-DP) [10], which is further converted by cellular kinases into the triphosphate derivate (ACV-TP) [11]. ACV-TP has a dGTP-like structure and is incorporated by the viral DNA polymerase into the viral DNA, causing destabilization of the DNA and ultimately inhibiting DNA elongation [9,12,13,14]. This strategy of selectively targeting viral processes, specifically the activation of acyclovir by viral thymidine kinase (TK) and its preferential concentration within infected cells, ensures minimal collateral damage to healthy cells. This mechanism allows acyclovir to effectively impede the proliferation of the herpesviruses, aiding the immune system in managing infection. While it does not eradicate herpesviral infections, acyclovir significantly mitigates the severity and duration of outbreak episodes.

Although acyclovir is primarily used in human medicine, it has also been tested both in vitro and in vivo against herpesviral infections in tortoises and turtles [15,16], as well as against some economically important fish herpesviruses, namely Cyprinid herpesvirus 3 (CyHV-3), colloquially known as koi herpesvirus, and Ictalurid herpesvirus 1, also known as channel catfish virus (IcHV-1) [17,18,19,20,21,22].

The European catfish virus (ECV) and Frog virus 3 (FV3) belong to the genus *Ranavirus* within the family *Iridoviridae* [23], while Ictalurid herpesvirus 2 (IcHV-2) belongs to the genus *Ictavirus* (the family *Alloherpesviridae*) [24]. Both virus families contain pathogens infecting fish and amphibians (Anamnia) and amongst them emerging aquatic viruses of rising concern, as thoroughly reviewed recently [25]. Both ranaviruses and herpesviruses have double-stranded DNA (dsDNA) genomes encoding TK genes. ECV and IcHV-2 have been isolated from fish species belonging to the order Siluriformes and are known to cause serious infections with high mortality [26,27]. FV3 has been isolated from several amphibian species [28] and causes severe infections [29,30]. While ECV and IcHV-2 can cause significant losses in aquaculture and FV3 can cause major damage in amphibian populations, there are no practical cures or effective therapies for these viral infections. Although silver nanoparticles have proven to be effective against ECV and IcHV-2 in vitro [31], preventive measures remain the only effective method to reduce the risk of infections [32].

In this study, we conducted molecular modeling and docking simulations to predict and elucidate the potential effectiveness of acyclovir against two ranaviruses with veterinary and ecological importance: ECV and FV3. IcHV-2 was used as a positive control. Subsequently, experimental assessments were performed to evaluate the efficacy of acyclovir against these viruses.

## 2. Materials and Methods

### 2.1. In Silico Protein Modeling

In silico protein modeling involved the deoxyribonucleoside kinase from IcHV-2, with accession number YP_009447832.1; the ECV deoxynucleoside kinase (AMZ04856.1); and the FV3 deoxynucleoside kinase (YP_031664.1). These proteins were modeled using AlphaFold2 (https://github.com/google-deepmind/alphafold, accessed on 3 April 2024), a state-of-the-art deep learning software that automates the prediction of protein structures with remarkable accuracy, as reported by [33]. For each viral protein, five models were generated and evaluated using AlphaFold2’s protein structure quality assignment module to ensure their reliability. The highest-quality model for each protein was then chosen for further refinement. This subsequent refinement process was carried out using the Schrödinger Suite (https://www.schrodinger.com/, accessed on 3 April 2024), which was employed to resolve any steric conflicts among the side-chain atoms, thereby ensuring the structural integrity of the proteins [34]. Pairwise protein sequence alignments among the examined TKs were calculated with the EMBOSS Needle tool (https://www.ebi.ac.uk/jdispatcher/psa/emboss_needle, accessed on 3 April 2024) [35]. For a visual representation of the modeled structures, we employed VMD version 1.9.3 (https://www.ks.uiuc.edu/Research/vmd/, accessed on 3 April 2024) [36] to create detailed molecular graphics.

### 2.2. Acyclovir Docking

In our molecular docking studies, we utilized the crystal structure of Human alphaherpesvirus 1 (HSV-1) TK in complex with the antiviral drug acyclovir (PDB ID: 2KI5) as a reference. The objective was to evaluate the binding affinity of acyclovir with the kinase enzymes of IcHV-2, ECV, and FV3. For this purpose, the Glide ligand docking module within the Schrödinger Suite [34] served as the primary tool. The molecular conformation of acyclovir, as determined by its X-ray crystallographic structure (PDB ID: 2KI5), was utilized as the ligand input for the docking simulations. Docking grids were prepared using the Receptor Grid Generator module of Schrödinger’s Glide, ensuring that acyclovir was accurately docked into both the reference structure and the models generated for the IcHV-2, ECV, and FV3 kinases. These docking procedures were carried out with the standard precision settings, and the ligand’s conformational flexibility was fully accounted for during sampling. Subsequently, the best ranking docking poses, indicative of the most favorable binding affinities, were identified and selected for further comparative analysis. The Ligand Interaction Diagram [34] tool was employed to elucidate critical interactions between the ligand and the proteins.

### 2.3. Cells and Viruses

Epithelioma Papulosum Cyprini (EPC; ATCC CRL-2872) cells were grown and handled as described in detail previously [31]. IcHV-2 originates from Italy [27], while the ECV strain (14612/2012) was isolated from the brown bullhead in Hungary [37]. The Frog Virus 3 strain Granoff (FV3, ATCC VR-567) was originally isolated from a leopard frog (*Lithobates pipiens*) in the USA in 1965 [38] and was propagated on various fish and reptilian cell lines before it was added to our collection by courtesy of Rachel Marschang (University of Hohenheim, Stuttgart, Germany) [39]. All three viruses were cultivated in the EPC cell line at 25 °C. The tissue culture infectious dose (TCID_50_/mL) was calculated by the Reed and Muench method [40].

### 2.4. Acyclovir Solutions

Lyophilized acyclovir powder (Sigma-Aldrich, St. Louis, MO, USA) was diluted in the fully supplemented EMEM used to grow the EPC cells and then filtered through a 0.45 µm membrane filter (Merck Millipore, Burlington, MA, USA). The following concentrations were prepared for the experiments: 3200 µM, 1600 µM, 800 µM, 400 µM, 200 µM, 100 µM, and 50 µM.

### 2.5. Cytotoxicity Assay

In brief, 96-well plates were seeded with the EPC cells at a density of 2 × 10^4^ cells/well and incubated for 24 h at 25 °C. The growth medium was then replaced with acyclovir-containing medium at concentrations of 3200 µM, 1600 µM, 800 µM, 400 µM, 200 µM, 100 µM, and 50 µM. The cells were incubated at 25 °C for three days, after which the cytotoxic effect of acyclovir was determined by the Vybrant^®^ MTT Cell Proliferation Assay Kit (Invitrogen, Carlsbad, CA, USA).

### 2.6. In Vitro Assays for Acyclovir and Virus Interactions

The EPC cells were seeded into 96-well plates (2 × 10^4^ cells/well) and incubated overnight at 25 °C. Virus titration (using 10-fold serial dilutions) was then carried out using acyclovir concentrations of 3200 µM, 1600 µM, and 800 µM in preliminary experiments. Subsequently, only the 800 µM concentration was used to determine the antiviral effect of acyclovir. The titers were read after 10 days of incubation at 25 °C.

The EPC cells were also seeded in 24-well plates (2 × 10^5^ cells/well) and incubated overnight at 25 °C. The following day, the cells were infected with the viruses at a MOI (multiplicity of infection) of 0.05 in EMEM medium alone (control) or EMEM containing 800 µM of acyclovir (treated). The plates were incubated for 48 h at 25 °C. Afterwards, the plates were frozen at −20 °C, followed by nucleic acid extraction and qPCR analysis.

### 2.7. Quantitative PCRs

Extraction of viral DNA was carried out with the Viral Nucleic Acid Extraction Kit II (Geneaid, New Taipei City, China) according to the manufacturer’s instructions. The extracted DNA was stored at −20 °C. Quantitative PCRs (qPCRs) were used to determine the relative amount of viral DNA in the treated and control groups. The qPCRs were performed in a Bio-Rad^®^ Real-Time PCR System instrument (Bio-Rad, Hercules, CA, USA) using the SensiFast^TM^ SYBR Hi-ROX Kit (Bioline, London, UK). The PCR mixture contained 10 µL of 2xSensifast SYBR mix, 0.8 µL of forward and reverse primers, 6.4 µL pf distilled water, and 2 µL of the target DNA. The final 20 µL mixture was used in a program that included an initial denaturing step at 95 °C for 3 min, followed by 40 cycles of 95 °C for 5 s and either 65 °C or 72 °C for 30 s. All the qPCRs were performed in duplicate, and the beta-actin gene of the EPC cells was used as an internal standard. The resulting qPCR data were analyzed by the Bio-Rad CFX Maestro Software. During the experiment, ranavirus major capsid protein forward and reverse primers were used for both the ECV and FV3 viruses [26]. The primer pairs for the amplification of IcHV-2 and β-actin of the EPC cells were described earlier [31,41]. The raw data on the cell viability assay and the qPCR are available upon request.

### 2.8. Statistical Data Analysis

Significance was calculated using two-sample, two-tailed Student’s *t*-tests. *p* values of <0.05 were considered to be significant. The results presented are representative data from a minimum of three separate experiments, with at least three experimental replicates per group. All error bars represent the standard error of the mean (SEM).

## 3. Results

### 3.1. Acyclovir Docking

**Figure 1:**
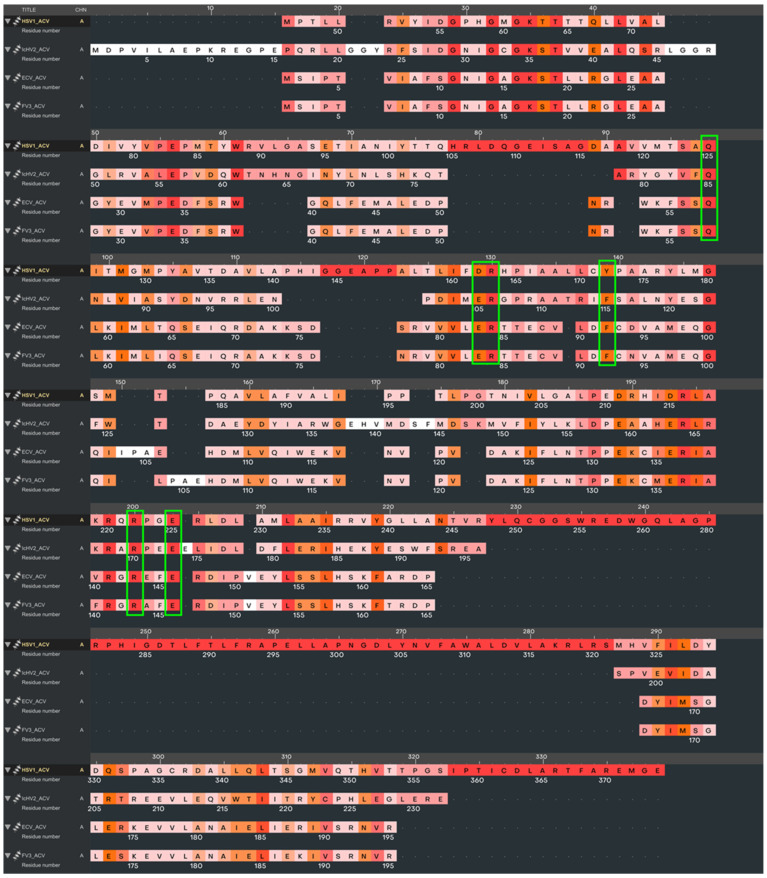
Superposition of protein structures and multiple sequence alignment of studied viral thymidine kinases. Active site residues are marked with green rectangles. Within the sequence alignment, amino acid residues are color-coded based on their similarity: red indicates high similarity, orange denotes medium similarity, and pink and white represent low similarity, facilitating an intuitive understanding of the conservation across the sequences.

**Figure 2:**
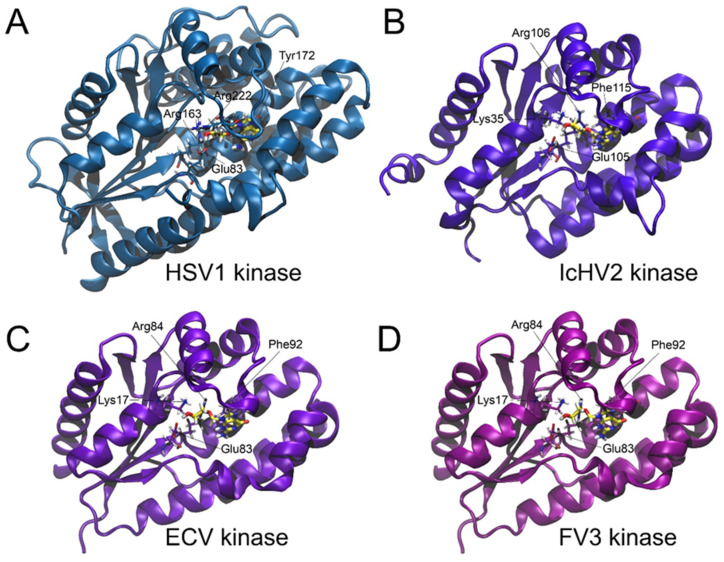
Structural comparison of thymidine kinases from Human alphaherpesvirus 1 (HSV-1) (A), Ictalurid herpesvirus 2 (IcHV-2) (B), European catfish virus (ECV) (C), and Frog virus 3 (FV3) (D) upon binding with acyclovir. Each kinase structure is depicted as a cartoon representation, with the acyclovir molecule and the critical active site residues highlighted in a stick representation.

**Figure 3:**
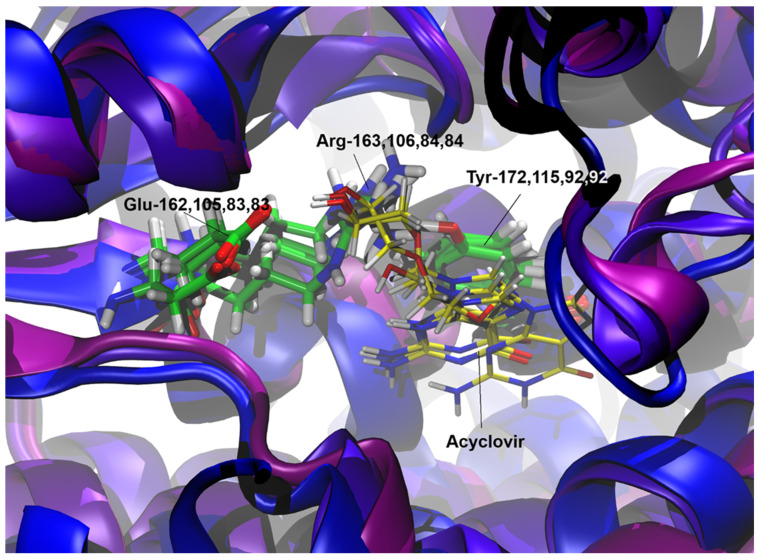
Structural superposition of thymidine kinase (TK) enzymes from Human alphaherpesvirus 1 (HSV-1), Ictalurid herpesvirus 2 (IcHV-2), European catfish virus (ECV), and Frog virus 3 (FV3). The superposition highlights the conserved structural fold despite the low sequence identity among the four TKs. Active site residues critical for the binding of acyclovir are shown in a stick representation, with HSV-1 (Tyr172, Phe115, and Phe92), IcHV-2 (Phe115, Glu105, and Arg106), ECV (Phe92, Glu83, and Arg84), and FV3 (Phe92, Glu83, and Arg84) indicated. The proposed acyclovir binding site is emphasized, illustrating the structural alignment and π-π stacking interactions essential for substrate coordination.

The validation of the docking procedure was confirmed by the alignment of the acyclovir molecule with its counterpart in the HSV-1 TK as described in the X-ray crystal structure reported by [42]. A comparative analysis revealed that the amino acid sequence identities and similarities between the reference HSV-1 TK and the thymidine kinases of the viruses under study (IcHV-2, ECV, and FV3) were notably low, as summarized in Table 1. Structural alignment was conducted to reveal the active site positions within the viral thymidine kinase models (IcHV-2, ECV, and FV3) using the HSV-1 structure as the reference framework. The structural superimposition based on multiple amino acid sequence alignments of the TKs is illustrated in Figure 1, highlighting the conservation of nucleotide binding sites. Notably, the π-π stacking interactions that coordinate the aromatic ring components of the nucleotide or the acyclovir molecule are conserved across the thymidine kinases: Tyr172 in HSV-1, Phe115 in IcHV-2, and Phe92 in both ECV and FV3 TKs. Additionally, essential basic and acidic residues that facilitate the orientation of the substrate’s free hydroxyl group before phosphorylation were identified. Comprehensive molecular graphics and a detailed representation of the structure-based multiple sequence alignment are provided in Figure 1 and Figure 2. Molecular docking simulations, as summarized in Table 2 and Figure 2, demonstrated that the acyclovir molecule is capable of binding to the active sites of all the viral thymidine kinases investigated in an orientation conducive to phosphorylation. This orientation is crucial, as phosphorylation represents the initial step in the mechanism of action of acyclovir. To further elucidate the structural similarities between the thymidine kinases (TKs) from HSV-1, IcHV-2, ECV, and FV3, we performed a structural superposition analysis. Despite their low sequence identity, the TKs from these viruses exhibit a remarkably similar fold. This structural conservation is particularly evident at the active site, where critical residues like Tyr172 in HSV-1, Phe115 in IcHV-2, and Phe92 in both ECV and FV3 are preserved. These residues are involved in π-π stacking interactions that stabilize the aromatic ring of the nucleotide or the acyclovir molecule, facilitating its proper orientation for phosphorylation. The superposition of these structures, especially at the proposed acyclovir binding site, underscores the potential of acyclovir to inhibit the TKs across these diverse viruses effectively. The superimposed structures at the acyclovir binding site are depicted in Figure 3, which clearly shows the alignment of the critical active site residues across the studied TKs. This finding supports the hypothesis that acyclovir can act as a broad-spectrum antiviral agent by targeting structurally conserved sites in viral TKs.

### 3.2. Cytotoxicity Assays

There was no observable alteration in the cell morphology in the treated cells. The MTT assay did not show any significant differences in the viability of the cells treated with acyclovir at any concentration compared to the control cells (Table 3).

### 3.3. In Vitro Assays

**Figure 4:**
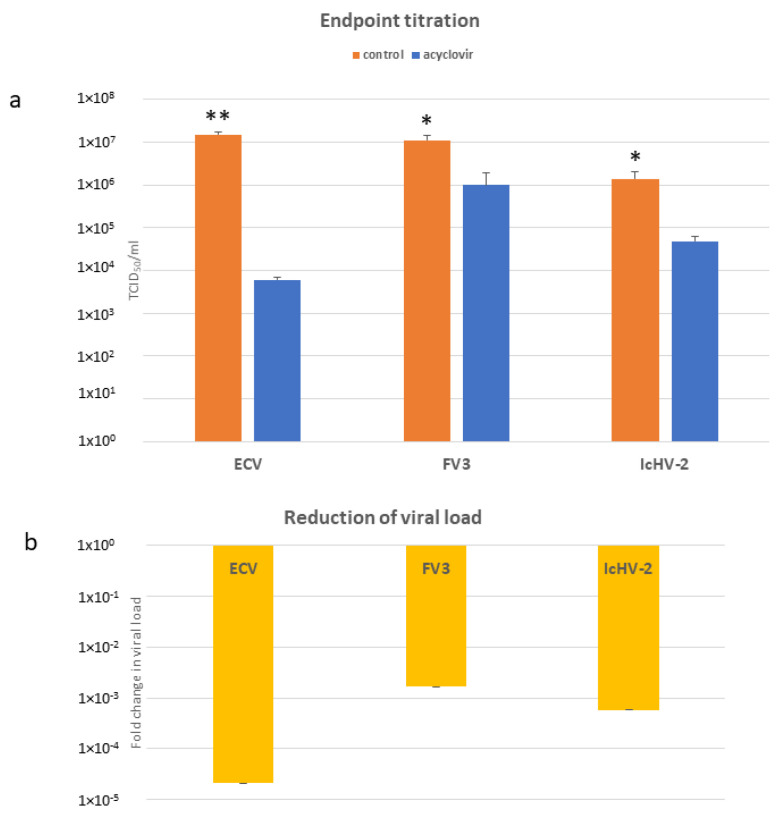
(**a**) TCID_50_/mL values after 10 days of incubation with 800 µM. Data from three separate experiments are shown as the mean +/− SEM. * *p* < 0.05, ** *p* < 0.01 vs. matched controls. (**b**) Relative amount (ΔΔCt) of the viral DNA (viral load) assessed by qPCR, Ct values were normalized against beta-actin of the EPC cells. The results are presented as a fold change compared to the mean of the control group (non-treated cells). Values are expressed as the mean +/− SEM.

In the preliminary experiment, acyclovir at concentrations of 3200, 1600, and 800 µM reduced the viral loads of ECV, FV3, and IcHV-2 at the same rate. Crystallization of acyclovir was observed in the wells with the highest concentration (3200 µM). In subsequent experiments, we only utilized the lowest concentration of the antiviral compound that remained effective. The 800 µM acyclovir concentration significantly reduced the viral load of all three viruses (Figure 4a). The largest decrease was observed for ECV (2500-fold), followed by the control virus, IcHV-2 (30-fold), and lastly, FV3 was found to be reduced 11-fold.

### 3.4. qPCRs

Similarly to the endpoint titration, the 800 µM acyclovir concentration significantly reduced the viral load of all three viruses (Figure 4b). The trends in the titration assays and in these qPCR experiments are alike: the viral DNA load of ECV was reduced the most (10^5^-fold), followed by IcHV-2 (10^4^-fold), and the least reduction was observed for FV3 (10^3^-fold).

## 4. Discussion

Aquaculture faces considerable risks and losses due to bacterial, fungal, parasitic, and viral infections. While several antibacterial and antiparasitic treatments are known and used in fish farming, effective, widely used, and direct antiviral therapies do not yet [43]. In recent decades, acyclovir has been used against herpesvirus infections in human medicine [44] and in veterinary medicine for avian and mammalian species as well [45,46]. Regarding poikilothermic vertebrates, acyclovir has been tested under experimental conditions against herpesviruses affecting aquaculture [17,19,20,21,22]. Additionally, the pharmacokinetics of acyclovir have been investigated in turtle species [15], and its anti-herpesviral effects have been studied in tortoise species [16]. Moreover, acyclovir appears to inhibit the replication of the white spot syndrome virus (the family *Nimaviridae*), which causes significant losses in crayfish farming [47]. To the best of our knowledge, no publication has reported about the effect of acyclovir on ranaviruses (the family *Iridoviridae*), although it has been used anecdotally to treat iridovirus infections in chelonians [15].

In this experiment, we studied the antiviral activity of acyclovir against ranaviruses of veterinary importance. ECV causes outbreaks with high mortality rates in different catfish species [48,49], and FV3 has also been reported to cause significant damage to various amphibian species [29,30]. As a positive control, we chose IcHV-2, a close relative of IcHV-1 [50], the replication of which has been experimentally inhibited by acyclovir in previous studies [18,19].

Initially, the amino acid sequences and structures of the ranaviral TKs were modeled in silico and compared to those of herpesviruses. The active sites of the enzymes were further studied, and the docking scores and binding energies of acyclovir to the viral thymidine kinases were evaluated. After confirming the potential for phosphorylation of the hydroxyl group on the acyclovir molecule following binding to the TKs, we evaluated the safe concentration of acyclovir in the EPC cells. It proved to be safe up to 3200 µM, which was the threshold of acyclovir’s solubility in the medium. In previous publications, the safe concentration of acyclovir was found to vary significantly, with one study reporting it to be 66.67 µM [17], while another found it to be 1500 µM [21] in the same cell line (KF-1). Following the cytotoxicity assays, higher concentrations of acyclovir (3200 µM, 1600 µM, and 800 µM) were tested in a preliminary experiment. The lowest concentration that remained effective, 800 µM, was chosen for subsequent assays, which is still only half of the concentration used in previous studies [21]. At this dose, acyclovir significantly decreased the viral load/titer of all three viruses. Acyclovir was most effective against ECV, followed by IcHV-2, and least effective against FV3, though still promising. The rate of the reduction in the viral load/virus titer is encouraging. In previous in vitro studies using 66.67 µM of acyclovir against CyHV-3, a 67–93% reduction in the viral load and a 25–66% decrease in the plaque number were observed in KF-1 and CCB cells [17]. In a subsequent experiment, a 98% reduction was achieved using higher concentrations (222.02 and 444.04 µM) [51]. In another experiment, 10 µM of acyclovir reduced the plaque number of IcHV-1 by 99% in BB cells [19].

The molecular modeling and docking results provided a strong theoretical foundation for the experimental work. The docking studies predicted that acyclovir binds effectively to the TKs of IcHV-2, ECV, and FV3, which was supported by subsequent in vitro experiments showing significant reductions in the viral loads. The alignment of the docking results with the experimental outcomes underscores the potential of acyclovir as a viable antiviral agent against ranaviruses, offering a promising avenue for developing effective treatments for viral infections in aquaculture.

The goal of our experiments was to investigate the impact of acyclovir on ranaviruses (*Iridoviridae*) in vitro. We can conclude that acyclovir is a potent inhibitor of ranavirus replication in cell culture conditions. These first in vitro results against this important group of viruses are encouraging to pursue to study the applicability and efficacy of acyclovir against ranaviral diseases in the future further.

## Figures and Tables

**Table 1:** Comparative analysis of pairwise amino acid sequence identities and similarities among studied viral thymidine kinases.

	Reference	HSV-1		IcHV-2		ECV		FV3	
Query		Identity %	Similarity %	Identity %	Similarity %	Identity %	Similarity %	Identity %	Similarity %
HSV-1				16.6	24.0	12.5	19.9	11.2	16.9
IcHV-2		16.1	22.4			21.5	37.2	21.1	36.4
ECV		12.5	19.9	21.5	37.2			93.8	96.9
FV3		11.2	16.9	21.1	36.4	93.8	96.9		

**Table 2:** Docking scores and binding energies of acyclovir to viral thymidine kinases. Phosphorylation feasibility denotes the potential for phosphorylation of the hydroxyl group on the acyclovir molecule after binding. Only the best docking scores are presented in this table. Lower docking cores correspond to higher affinity. The protein data bank accession number is given in parentheses for the reference structure.

	Glide Score	Glide Emodel/(kcal/mol)	Phosphorylation Feasibility
HSV-1 kinase (2KI5)	−8.730	−71.527	Yes
IcHV-2 kinase	−6.932	−64.247	Yes
ECV kinase	−7.239	−65.365	Yes
FV3 kinase	−7.299	−65.376	Yes

**Table 3:** The results of the viability assays. The cells were exposed to different acyclovir concentrations for three days. Viability was calculated as a percent of the control.

Concentration of Acyclovir (µM)	Cell Viability ± SEM (%)
3200	103.373 ± 1.58
1600	94.35 ± 1.47
800	102.70 ± 1.41
400	110.17 ± 3.45
200	110.68 ± 2.27
100	104.99 ± 1.48
50	113.00 ± 1.92

## Data Availability

All raw data are available upon reques.
